# Editorial and Miscellaneous

**Published:** 1873-10

**Authors:** 


### PART III.

### EDITORIAL AND MISCELLANEOUS.

*To our Subscribers.*

☞ LOOK OUT FOR THE RED CROSS!!

☞ Many of the subscribers for the RECORD have not paid the subscription price—$2.50. Do not delay this duty, friends. Our Publishing House having changed hands recently, the RECORD will, commencing with this number, be published by V. P. SISSON & Co., Proprietors of the "Economical Book & Job Printing House," of Atlanta. This change has necessarily caused delay in the appearance of the present issue. ☞ It also compels us to urge all who are in arrears for subscription to oblige us by *promptly* forwarding their dues. All who have or shall remit their dues will be credited and payment acknowledged in the next number. If we should fail to credit them in that issue of the journal, they will please inform us. All mistakes will be corrected with pleasure. Look out for the red cross—we are looking for the money!

☞ We can furnish a few new subscribers with the back numbers, if they wish to have this volume complete. It will be invaluable as a book of reference.

☞ All subscribers, and others, will please write their names, P. O., County and State plainly. ☞ Remember, this is now required by the Postmaster General.

☞ Should any mistake occur either in name or post - office, or should the RECORD fail to reach its destination, please inform the editors, who will have matters righted without delay.

☞ We invite our readers and friends every where to send us communications. Overhaul your case-books and send us your experience. Our columns are open to all.

☞ We will take the liberty to cull from private letters such extracts as may be deemed of special importance in promoting the interests of the profession.

☞ Dr. W. T. Taylor, of Kentucky, writes: "I will be one of two hundred who will send five new subscribers, each accompanied by the cash, between this and Christmas."

☞ The above proposition we place before our readers, and pledge ourselves (if two hundred of our subscribers will agree to pledge themselves to send us five new subscribers each by the first of January) to enlarge the size of the journal without additional cost, and to give a premium (in books, instruments or anything else desired) to the value of seventy-five dollars to the person getting the largest number of names over five, fifty dollars to the next, and twenty-five to the next. If fifty will send us five new subscribers, the one that sends the largest number over five shall have a premium of his own choosing worth twenty-five dollars. Go to work, friends; you have from now until the first of January to get up your list of names for this volume and the next, counting from the issuing of the August number, or the first of September.

☞ In addition to the premiums already offered, each new subscriber will be entitled to Wood's Household Magazine and the superb chromo—the Yosemite Valley, 14x20—for $3.30. So, for a small sum, the RECORD, a fine LITERARY MONTHLY and a *magnificent chromo* may be had.

Any one sending us three subscribers ($7.50) will be entitled to POWELL'S FORMULARY, or WOOD'S HOUSEHOLD MAGAZINE and CHROMO, gratis.

☞ Many who have not a complete volume of the RECORD would doubtless be pleased to have a bound copy at the expiration of the year. If so, we will furnish the entire volume ready-bound for your libraries, at cost, which we think will be about $4,00. Will our friends inform their medical friends of this?

*Proceedings of Societies.*

While our readers object to our filling our pages with uninteresting details of Medical Societies, yet we would be pleased to have our friends send us the interesting and instructive portions of the proceedings of their societies for publication. Friends, let us hear from you.

*Apologetic.*

Owing to a change of publishers, and from the fact that this number was passed through its publication rapidly, many typographical errors have disfigured it. Dr. T. Curtis Smith will pardon the "F" in his name.

*Powell's Pocket Formulary.*

On another page we present the contents of the above named *Formulary,* which will shew, at a glance, its character, scope and design. This is done because we have offered the *Formulary* as one of the premiums to subscribers. All persons who have sent us two (2) subscribers, by sending one more will be entitled to a copy gratis, when we are notified of the fact. All who will send us, from the September number two (2) new subscribers, will be entitled to a copy free.

*Our Exchanges: Please Take Notice.*

We have regularly and faithfully mailed the RECORD to all exchanges, but many of them come to us so irregularly, or fail to come altogether, that we beg them to look into this and rectify it. Doubtless this resulted from the change of name. We ask all who get our journal to do us the very special favor to see that the RECORD is placed upon their exchange list.

*Errata.*

In the article on "Tape Worm," page 610, in the prescription, oil of cinnamon should read : gtt. ii.

*Take Notice.*

We have not, nor will we in the future insert advertisements of druggists and others whom we cannot endorse and recommend to our readers as worthy of their confidence and patronage as first-class houses. We hope, therefore, that our friends throughout the country will request their home druggists to keep on hand the drugs, preparations, etc., of these houses for their use, in order to carry out the design we have in view—to-wit, that the profession may command pure medicines for their prescriptions.

Communications are solicited from all professional gentlemen every where. Send your articles along. The present number shows what our friends can do when they try. Friends! *write!* WRITE!! WRITE!!!

*Yellow Fever.*

As our readers are aware, Yellow Fever has prevailed in many cities and towns of the South and South-West, of appalling and unusual malignancy. At certain points fever has prevailed the exact nature of which has caused much diversion of sentiment among the resident physicians. We have received a report of a committee of Physicians of Houston and Galveston, Texas, who were appointed to determine the character of the fever prevailing at Calvert, Texas. The majority of the committee believed that yellow fever prevailed. We will add that the committee was composed of some of the ablest medical men of Texas. We were pleased to see that Dr. Kilpatrick, one of our Texas associates, was requested to meet with them. We would be glad to hear from any or all of these gentlemen upon any questions touching the interests of the profession.

*The Trustees of Jefferson Medical College, Philadelphia,*

Having requested Prof. Joseph Pancoast to withdraw his intended resignation of the chair of Anatomy, he has, we learn, complied with the request, and will deliver the course during the ensuing session.

*Specialties and Subdivision of Practice.*

Dr. Robert Barnes, of London, in a lecture at the Royal College of Physicians, took occasion to speak of the subdivision of practice which is getting to be so general, especially in metropolitan cities. As an illustration of the thing, he remarked that the public seems to grow less and less reasonable upon this subject every day.

"I have recently been honored by a visit from a lady of typical modern intelligence, who consulted me about a fibroid tumor of the uterus; and lest I should stray beyond my business, she was careful to tell me that Dr. Brown-Sequard had charge of her nervous system; that Dr. Williams attended to her lungs; that her abdominal organs were entrusted to Sir William Gull; that Mr. Spencer Wells looked after her rectum; and that Dr. Walshe had her heart. If some adventurous doctor should determine to start a new specialty, and open an institution for the treatment of diseases of the *umbilicus*—the only region which, as my colleague, Mr. Simon, says is unappropriated—I think I can promise him more than one patient."—*August No. London Lancet.*

